# Existe Relação entre Atividade Física no Tempo Livre e Incidência de Hipertensão Arterial?

**DOI:** 10.36660/abc.20240318

**Published:** 2024-07-23

**Authors:** Jacqueline Lima, Leandro Franzoni, Elren Passos Monteiro

**Affiliations:** 1 Universidade Federal do Pará - Campus Castanhal Castanhal Brasil Programa de Pós-graduação em Ciências do Movimento Humano - Universidade Federal do Pará - Campus Castanhal, Castanhal – Brasil; 2 Universidade Federal do Rio Grande do Sul Porto Alegre RS Brasil Programa de Pós-Graduação em Ciências da Saúde: Cardiologia e Ciências Cardiovasculares – Universidade Federal do Rio Grande do Sul, Porto Alegre, RS – Brasil

**Keywords:** Doenças Cardiovasculares, Exercício Físico, Hipertensão

As doenças cardiovasculares (DCVs) são um grupo de doenças ligadas aos vasos sanguíneos, ao coração e aos tecidos adjacentes que impactam na saúde e na vida normal, sendo que é uma doença que possui uma das mais altas taxas de morbimortalidade no mundo.^1,2^ Em 2019, a maior causa de morte no mundo foram as DCVs, responsáveis por 16% do total de mortes.^2^ No mesmo ano, apresentou um total de mortes de 8,9 milhões ao redor do mundo.^2^

As DCVs são a principal causa de morte, hospitalizações e atendimentos ambulatoriais em todo o mundo, inclusive em países em desenvolvimento como o Brasil.^1,3^ Assim como, as DCVs, a hipertensão arterial (HA) é o principal fator de risco para morbidade, mortalidade e incapacidade por DCVs.^1^

A HA é uma doença crônica não transmissível definida por níveis pressóricos que a categorizam, assim como, é uma condição multifatorial, que depende de fatores genéticos, epigenéticos, ambientais e sociais, caracterizada fisiologicamente por elevação persistente da pressão arterial (PA).^1^ Ela pode ser evitável ou adiada por um conjunto de intervenções preventivas, entre as quais incluem a redução da ingestão de sal, uma dieta rica em frutas e vegetais e exercício físico.^4^ Estudos anteriores sugeriram que a atividade física no tempo livre (AFTL), estava inversamente associada ao risco de HA.^3,4^ Assim, a AFTL tem sido recomendada como um estilo de vida preventivo para HA.^3^

Diante disso, nesta edição dos Arquivos Brasileiros de Cardiologia, Souza et al.,^5^ investigaram de forma longitudinal, a associação entre as mudanças na AFTL e a incidência de HA em participantes do ELSA-Brasil (Estudo Longitudinal de Saúde do Adulto). O ELSA-Brasil é um grande estudo de coorte multicêntrico, que tem como foco investigar a incidência e a progressão da obesidade, diabetes e DCVs, em adultos brasileiros com idade entre 35 e 74 anos.^6,7^

O estudo analisou dados de 8.968 participantes, sendo 4916 mulheres e 4052 homens, em dois períodos distintos: da linha de base (2008-2010) e acompanhados até o primeiro segmento (2012-2014).^8^ Foram selecionados indivíduos com PA normal (normotensos) e excluiu qualquer pessoa com diagnóstico de HA ou que estivesse sob uso de medicamentos para o tratamento da HA. Todos os participantes responderam os questionários sobre avaliação da AFTL (Questionário Internacional de Atividade Física -IPAQ- versão longa), que é composto por questões relativas à frequência e à duração de atividades físicas (caminhada - moderada e vigorosa) desenvolvidas no trabalho, no deslocamento, nas atividades domésticas e no tempo livre.^9^

Como resultado, Souza et al.,^5^ identificaram que a incidência de HA no primeiro segmento foi de 16,9%, sendo 12,8% entre as mulheres e de 21,9% entre os homens, indicou também associação estatística entre AFTL e HA em participantes muito ativos: risco de HA reduzido em 35% entre homens RR = 0,65 (IC 95% 0,50-0,86) e risco de HA reduzido em 66% entre as mulheres RR = 0,34 (IC 95% 0,20-0,58).

Com tudo isso, os autores na discussão associam a AFTL, o grupo muito ativo fisicamente, a um menor risco de desenvolver HA ao longo do tempo. Ademais, as mulheres tiveram uma redução ainda mais acentuada no risco em comparação aos homens. Contudo, isso não significa que a AF moderada seja ineficaz na prevenção da HA,^8^ visto que realização de AFTL com intensidade moderada a vigorosa já se mostrou eficiente para prevenção de condições como as DCVs e até diabetes.

Os resultados deste estudo destacam o papel crucial da AFTL na redução do risco de HA, especialmente quando praticada de forma consistente. Estes achados sublinham a importância de promover um estilo de vida ativo como medida preventiva contra a HA independentemente do sexo.^10,11^
