# Supplementary material for: Existe Relação entre Atividade Física no Tempo Livre e Incidência de Hipertensão Arterial?
Source: Arq Bras Cardiol. 2024 Jul 23;121(6):e20240318. [Article in Portuguese] doi: 10.36660/abc.20240318 (PMC11364446; doi:10.36660/abc.20240318)
Supplement: Translation

# Translation: Is There a Relationship Between Physical Activity in Free Time and the Incidence of High Blood Pressure?

**DOI:** 10.36660/abc.20240318i

**Authors:** Jacqueline Lima, Leandro Franzoni, Elren Passos Monteiro

**Affiliations:** 1 Castanhal Brazil Programa de Pós-graduação em Ciências do Movimento Humano - Universidade Federal do Pará - Campus Castanhal, Castanhal – Brazil; 2 Porto Alegre RS Brazil Programa de Pós-Graduação em Ciências da Saúde: Cardiologia e Ciências Cardiovasculares – Universidade Federal do Rio Grande do Sul, Porto Alegre, RS – Brazil

**Keywords:** Cardiovascular Diseases, Exercise, Hypertension

Cardiovascular diseases (CVDs) are a group of diseases linked to blood vessels, the heart, and adjacent tissues that impact health and normal life, and it is a disease that has one of the highest morbidity and mortality rates in the world.^1,2^ In 2019, the biggest cause of death in the world was CVDs, responsible for 16% of total deaths.^2^ In the same year, there were a total of 8.9 million deaths around the world.^2^

CVDs are the main cause of death, hospitalizations, and outpatient care throughout the world, including in developing countries such as Brazil.^1,3^ Just like CVDs, arterial hypertension (AH) is the main risk factor for morbidity, mortality, and disability due to CVDs.^1^

AH is a chronic non-communicable disease defined by blood pressure levels that categorize it, as well as a multifactorial condition, which depends on genetic, epigenetic, environmental, and social factors, physiologically characterized by persistent elevation of blood pressure (BP).^1^ It can be preventable or delayed by a set of preventive interventions, including reducing salt intake, a diet rich in fruits and vegetables, and physical exercise.^4^ Previous studies have suggested that physical activity in free time (PAFT), was inversely associated with the risk of AH.^3,4^ Thus, PAFT has been recommended as a preventive lifestyle for AH.^3^

Therefore, in this edition of the *Arquivos Brasileiros de Cardiologia*, Souza et al.,^5^ investigated longitudinally, the association between changes in PAFT and the incidence of AH in participants of the ELSA-Brazil (Longitudinal Study of Adult Health). ELSA-Brazil is a large multicenter cohort study, which focuses on investigating the incidence and progression of obesity, diabetes, and CVD, in Brazilian adults aged between 35 and 74 years.^6,7^

The study analyzed data from 8,968 participants, 4916 women, and 4052 men, in two different periods: from baseline (2008-2010) and followed until the first segment (2012-2014).^8^ Individuals with normal BP (normotensive) were selected and anyone diagnosed with AH or who was taking medication to treat AH was excluded. All participants answered the questionnaires on assessment of PAFT (International Physical Activity Questionnaire -IPAQ- long version), which is composed of questions relating to the frequency and duration of physical activities (walking - moderate and vigorous) carried out at work while commuting, in domestic activities and in free time.^9^

As a result, Souza et al.,^5^ identified that the incidence of AH in the first segment was 16.9%, 12.8% among women, and 21.9% among men, also indicating a statistical association between PAFT and AH in very active participants: risk of AH reduced by 35 % among men RR = 0.65 (95% IC 0.50-0.86) and risk of AH reduced by 66% among women RR = 0.34 (95% IC 0.20-0.58).

With all this, the authors in the discussion associate PAFT, the very physically active group, with a lower risk of developing hypertension over time. Furthermore, women had an even more pronounced reduction in risk compared to men. However, this does not mean that moderate PA is ineffective in preventing AH,^8^ as performing PAFT with moderate to vigorous intensity has already been shown to be efficient in preventing conditions such as CVD and even diabetes.

The results of this study highlight the crucial role of PAFT in reducing the risk of AH, especially when practiced consistently. These findings highlight the importance of promoting an active lifestyle as a preventive measure against AH regardless of gender.^10,11^
